# Unexplained oral and extremity ulcerations in an infant

**DOI:** 10.1016/j.jdcr.2021.01.010

**Published:** 2021-01-19

**Authors:** Kristen Russomanno, Ashley DiLorenzo, Kara Simpson, Seth Berger, Kaiane Habeshian

**Affiliations:** a Department of Dermatology, Medstar Washington Hospital Center/Georgetown University Hospital, Washington, DC; b Department of Internal Medicine, Medstar Washington Hospital Center, Washington, DC; c Center for Genetic Medicine Research & Rare Disease Institute, Children's National Hospital, Washington, DC; d Division of Dermatology, Children's National Hospital, Washington, DC; e Department of Dermatology, George Washington University School of Medicine and Health Sciences, Washington, DC

**Keywords:** congenital insensitivity to pain, genetic diseases, hereditary sensory and autonomic neuropathies, pain insensitivity, pediatric dermatology, CIP, congenital insensitivity to pain, HED, hypohidrotic ectodermal dysplasia, HSAN, hereditary sensory and autonomic neuropathies, LCH, langerhans cell histiocytosis, OI, osteogenesis imperfecta

A 5-month-old healthy, developmentally appropriate male presented to the emergency department with a 2-month history of bleeding ulcerations of the lower lip and floor of the mouth, (Fig 1) and several healing ulcerations on the fingers and toes. The child appeared well and was in no distress on examination. Vital signs were normal. Bacterial, fungal, and enterovirus cultures from the mouth, as well as herpes simplex virus polymerase chain reaction and HIV and syphilis testing were negative. Complete blood count and comprehensive metabolic panel were unremarkable. Biopsies of the oral and digital ulcerations performed by otolaryngology and dermatology showed granulation tissue and epidermal spongiosis consistent with reactive changes (Fig 2).

**Fig 1:**
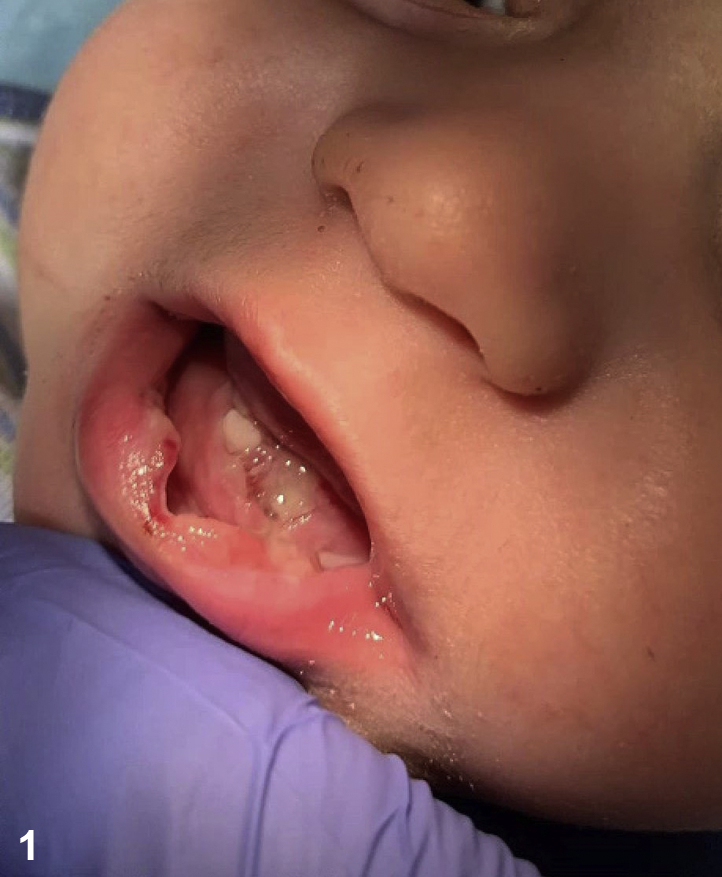

**Fig 2:**
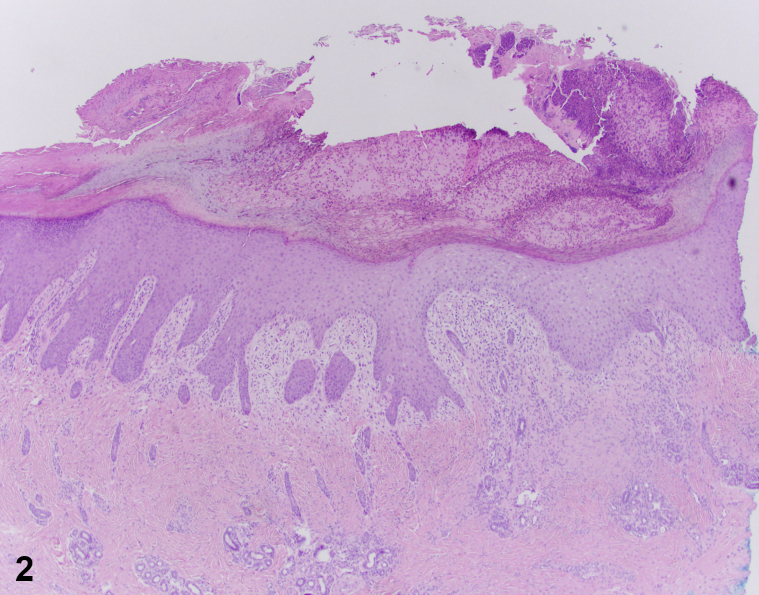

**Question 1: What is the most likely diagnosis?**

A. Child physical abuse
B. Congenital insensitivity to pain (CIP)
C. Langerhans cell histiocytosis (LCH)
D. Osteogenesis imperfecta (OI)
E. Lesch-Nyhan syndrome

**Answers:**

A. Child physical abuse – incorrect. Child abuse should be suspected as an etiology of unexplained injuries in an infant, and this was initially considered. Given this patient was well appearing and showed no sign of discomfort in association with his injuries, other diagnoses were more strongly considered.
B. CIP – correct. CIP belongs to a group of hereditary sensory and autonomic neuropathies (HSAN), of which there are several subtypes.^1^^,^^2^ It would be expected that pain, discomfort, and/or decreased oral intake would be present in the setting of a bleeding ulceration in the mouth. These features were not observed, and thus the diagnosis of CIP was considered and later confirmed by genetic testing.
C. LCH – incorrect. LCH may present with a wide range of mucocutaneous lesions, including ulceration of the oral and genital regions and thus the diagnosis was initially considered. Multisystem disease is common and may involve the bones, central nervous system, lymphatics, and lungs; however, this was not observed.^3^
D. OI – incorrect. OI may present with fractures that have occurred with minimal trauma, in addition to joint hypermobility, blue sclera, short stature, easy bruising, dentinogenesis imperfecta, and hearing loss.^3^ These features were not observed in this case. Furthermore, the patients affected by OI do experience pain in association with their injuries.
E. Lesch-Nyhan syndrome – incorrect. Lesch-Nyhan syndrome is an inherited inborn error of purine metabolism. Clinical features that overlap with CIP include progressive self-injurious behaviors. However, affected patients typically also experience hyperuricemia, severe developmental disability, dystonia, and chorea.^3^

**Question 2: The next best step to confirm the diagnosis is molecular genetic testing. Which of the following genes is *most likely* to have pathogenic variants in patients affected by this condition?**

A. SCN9A
B. HPRT1
C. COL1A1/2
D. PRDM12
E. BRAF

**Answers:**

A. SCN9A – correct. SCN9A encodes a voltage-gated sodium channel, and pathogenic variants of the gene result in CIP (HSAN type IID variant).^1^, ^2^, ^3^, ^4^, ^5^ Notably, there are multiple genetic abnormalities that lead to various different HSAN subtypes; however, in comparison to other genes, variations in SCN9A are more common.^3^^,^^5^
B. HPRT1 – incorrect. Pathogenic variants in HPRT1 are associated with Lesch-Nyhan syndrome, which is characterized by progressive self-injurious behavior, as well as hyperuricemia, severe developmental delay, and abnormal involuntary movements.^3^
C. COL1A1/2 – incorrect. COL1A1 and COL1A2 pathogenic variants are associated with OI, which, like CIP, can present with multiple fractures and other injuries. However, patients with OI experience pain from injuries and may have characteristic blue sclera, short stature, and joint hypermobility.^3^
D. PRDM12 – incorrect. Pathogenic variations in PRDM12 are associated with a rare variant of CIP (HSAN Type VIII). Ultimately, this patient had genetic testing showing a pathogenic variant of PRDM12, and the diagnosis was confirmed.^1^
E. BRAF – incorrect. Somatic BRAF variants, notably V600E, have been observed in tumor samples from patients with LCH. LCH was initially considered as a differential diagnosis given the ulceration in the mouth with radiographic changes that could not exclude mandibular bone erosion. In the absence of other characteristic clinical and histopathologic findings, however, LCH was excluded.

**Question 3: Patients with this condition manifest which of the following findings?**

A. Corneal injuries
B. *Staphylococcus aureus* infections
C. Hypotrichosis
D. Thin skin
E. A and B

**Answers:**

A. Corneal injuries – incorrect. Patients with CIP manifest *both* A and B. Corneal anesthesia and/or the absence of corneal reflexes is dependent on the associated pathogenic variant. However, all individuals affected by CIP are at risk of corneal injuries, and permanent corneal scarring may develop. Evaluation by ophthalmology at least annually is recommended.^3^^,^^4^
B. *Staphylococcus aureus* infections – incorrect. Patients with CIP manifest *both* choices A and B. Multiple HSAN subtypes are associated with an increased risk of *Staphylococcus aureus* infections, including HSAN VIII. Manifestations include recurrent soft tissue infections, abscesses, and osteomyelitis. Prompt investigation at any early sign of infection should be pursued.
C. Hypotrichosis – incorrect. Patients with hypohidrotic ectodermal dysplasia (HED) manifest hypotrichosis, abnormal dentition, and anhidrosis. Patients with CIP and other HSAN subtypes may be affected by anhidrosis and resultant thermal dysregulation like those affected by HED. However, patients with CIP and other HSAN subtypes lack the conical teeth and hypotrichosis characteristic of HED.
D. Thin skin – incorrect. Manifestations of OI include thin skin, blue sclerae, bone fragility, dentinogenesis imperfecta, hearing loss, easy bruising, and various neurologic and vascular abnormalities. Patients with OI and CIP may both present with bruising and fractures; however, a major distinguishing feature is normal pain perception in OI.
E. A and B – correct. Patients with CIP and other HSAN subtypes have increased susceptibility to *Staphylococcus aureus* infections, which may lead to recurrent soft tissue infections, osteomyelitis, and abscesses. Patients also are at an increased risk of corneal injuries, as above.

## Conflicts of interest

None disclosed.
